# Dr. M. F. Clausius

**Published:** 1916-05

**Authors:** 


**Dr. M. F. Clausius,** Buffalo 1891, died at his home in Siletz, Ore., Feb. 23, of cancer of the stomach, after an illness of about a year. He was born in Kruetinen, E. Prussia, March 11, 1852, and came to the U. S. in 1870. He was physician in the U. S. Indian Service. He bequeathed his medical library to the University of Buffalo.

